# Biometric Digital Health Technology for Measuring Motor Function in Parkinson’s Disease: Results from a Feasibility and Patient Satisfaction Study

**DOI:** 10.3389/fneur.2017.00273

**Published:** 2017-06-13

**Authors:** Georgia Mitsi, Enrique Urrea Mendoza, Benjamin D. Wissel, Elena Barbopoulou, Alok K. Dwivedi, Ioannis Tsoulos, Athanassios Stavrakoudis, Alberto J. Espay, Spyros Papapetropoulos

**Affiliations:** ^1^Apptomics Inc., Wellesley, MA, United States; ^2^Neuroscience Associates, Greenville Health System, Greenville, SC, United States; ^3^Gardner Family Center for Parkinson’s Disease and Movement Disorders, Department of Neurology, University of Cincinnati, Cincinnati, OH, United States; ^4^Department of Mathematics, City University London, London, United Kingdom; ^5^Department of Computer Science and Engineering, City University London, London, United Kingdom; ^6^Division of Biostatistics and Epidemiology, Department of Biomedical Sciences, Texas Tech University Health Sciences Center, El Paso, TX, United States; ^7^Department of Informatics and Telecommunications, Technological Educational Institute of Epirus, Epirus, Greece; ^8^Department of Economics, University of Ioannina, Ioannina, Greece; ^9^Department of Neurology, Massachusetts General Hospital, Boston, MA, United States

**Keywords:** motor symptoms, digital health, objective measures, Parkinson’s disease, smart tablet, digital biomarker

## Abstract

**Objectives:**

To assess the feasibility, predictive value, and user satisfaction of objectively quantifying motor function in Parkinson’s disease (PD) through a tablet-based application (iMotor) using self-administered tests.

**Methods:**

PD and healthy controls (HCs) performed finger tapping, hand pronation–supination and reaction time tasks using the iMotor application.

**Results:**

Thirty-eight participants (19 with PD and 17 HCs) were recruited in the study. PD subjects were 53% male, with a mean age of 67.8 years (±8.8), mean disease duration of 6.5 years (±4.6), Movement Disorders Society version of the Unified Parkinson Disease Rating Scale III score 26.3 (±6.7), and Hoehn & Yahr stage 2. In the univariate analysis, most tapping variables were significantly different in PD compared to HC. Tap interval provided the highest predictive ability (90%). In the multivariable logistic regression model reaction time (reaction time test) (*p* = 0.021) and total taps (two-target test) (*p* = 0.026) were associated with PD. A combined model with two-target (total taps and accuracy) and reaction time produced maximum discriminatory performance between HC and PD. The overall accuracy of the combined model was 0.98 (95% confidence interval: 0.93–1). iMotor use achieved high rates of patients’ satisfaction as evaluated by a patient satisfaction survey.

**Conclusion:**

iMotor differentiated PD subjects from HCs using simple alternating tasks of motor function. Results of this feasibility study should be replicated in larger, longitudinal, appropriately designed, controlled studies. The impact on patient care of at-home iMotor-assisted remote monitoring also deserves further evaluation.

## Introduction

Parkinson’s disease (PD) care has been limited by insufficient or poor quality patient data, inadequate, sporadic monitoring between in-office visits, delay in access to care, and infrequent visits (1). Closer patient monitoring and treatment “optimization” can lead to significant improvement in motor function and reduction in motor and non-motor fluctuations even before administration of experimental interventions as demonstrated in clinical trial settings (2).

Supplementing routine neurological evaluations with simple, user friendly, technology-enabled objective measures (TOMs) could increase precision in the assessment of motor changes in PD (3) and provide a more reliable and valid mechanism for patient monitoring at home. Such tools may strengthen the relationship between patients/caregivers and specialists and provide reliable, objective data on quality of life and use of health-care resources (1). Besides the quantification of the severity of symptoms, data captured by TOMs have the potential to track disease progression and provide insights of diagnostic and prognostic value for individual patients (4).

Most smartphones and tablets are equipped with state-of-the art sensing technology that includes touch screens, accelerometers, built-in cameras, and sensitive microphones capable of passively and actively capturing data for PD symptom monitoring. To date, despite the availability of multiple smart device applications for PD, only a small number have been subject of pilot or clinical testing (5–10). Some of the available applications reported in peer-reviewed literature include versions of one- or two-target finger tapping tests either without reporting results (6, 9, 10) or reporting data developed using different algorithmic approaches (8) or accelerometry-based technologies (5). Lack of reported data sets and standardization limits cross-study and cross-application comparisons. To our knowledge, this is the second detailed report of finger tapping data collected through a smart device in the peer-reviewed literature and the first that also includes pronation/supination hand tapping and addresses patient satisfaction. Lack of validation data sets and patient engagement prohibits leveraging the full potential of these technologies.

We sought to assess the feasibility and user satisfaction of objective quantification of motor function with iMotor and to explore its discriminatory performance in PD patients compared with healthy volunteers. Our specific objectives were to (1) assess the feasibility of capturing motor assessments interfacing with a touch-sensitive screen of a commercial-grade Android OS (Google, Inc.) 7-inch smart tablet in the clinical setting; (2) compare motor data between participants with PD and healthy controls (HCs); and (3) evaluate patient satisfaction with this application.

## Methods

We conducted an exploratory feasibility, single-center, cross-sectional study using iMotor (Apptomics, Wellesley, MA, USA) in individuals with PD and HCs. Participants gave informed, written consent for this study. All procedures were approved by the University of Cincinnati Institutional Review Board.

A movement disorder specialist at the University of Cincinnati evaluated all PD subjects. Unaffected family, caregivers, and friends of PD participants were recruited as HCs. Eligible participants were male or female between 18 and 75 years. PD subjects had a documented diagnosis of PD meeting UK Brain Bank criteria with Hoehn & Yahr (H&Y) stages I–IV. Key exclusion criteria included the following: (1) atypical Parkinsonian features; (2) any CNS disorder (other than PD for PD subjects); (3) evidence or history of clinically significant medical illness; and (4) evidence or history of condition that prohibited subjects from performing tasks outlined in the protocol.

Participants provided demographic information and medical history. The motor subscale of Movement Disorders Society version of the Unified Parkinson Disease Rating Scale (MDS-UPDRS-III) was also collected during a structured clinical evaluation.

**Figure 1:**
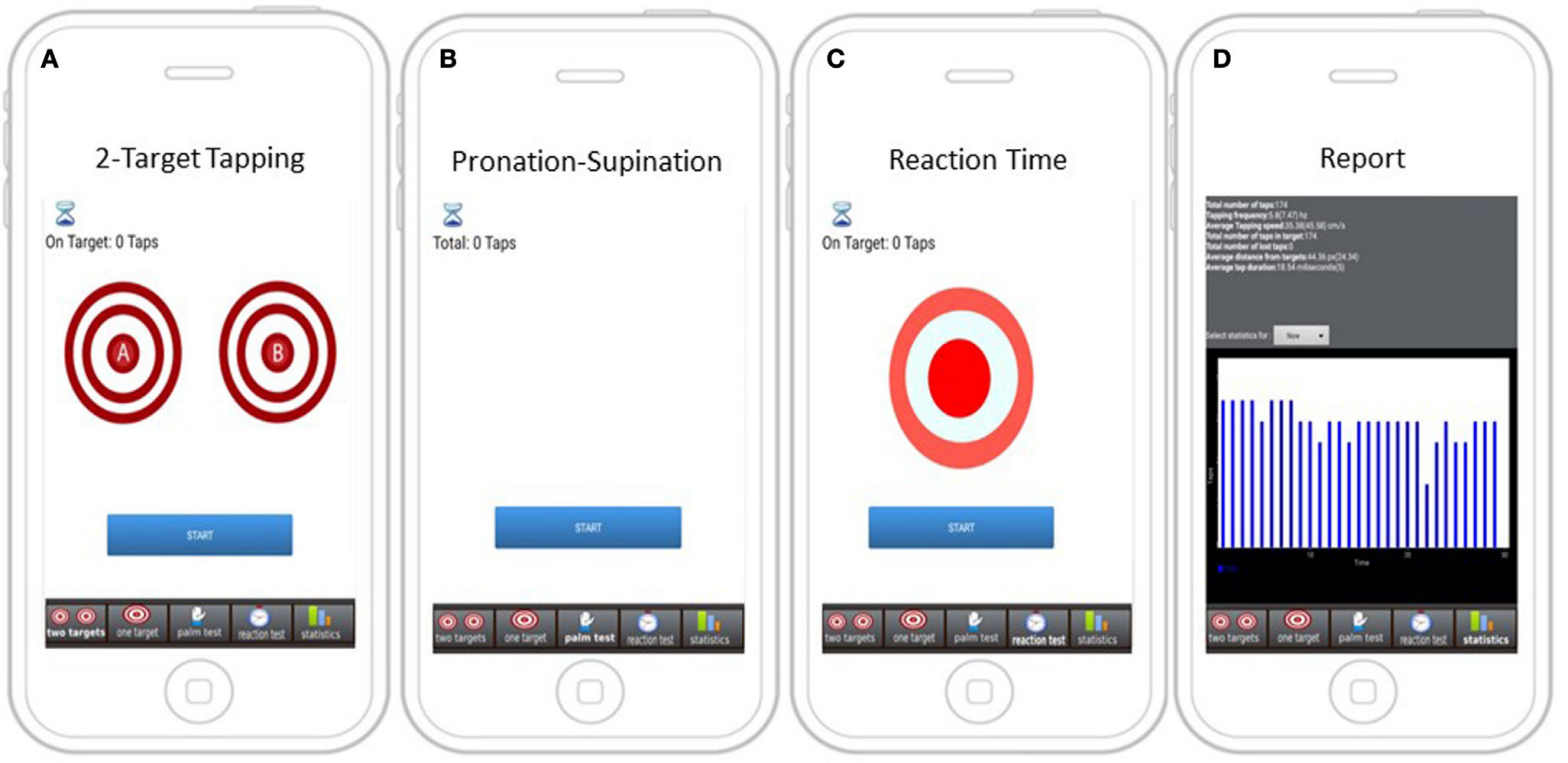
Screens with instructions for iMotor-based tasks. **(A)** Two-target test: participants tap alternatively with their index finger on the center of two concentric circles on the tablet screen. **(B)** Pronation–supination test: participants tap alternatively their palmar and dorsal surface of their hand on the tablet screen. **(C)** Reaction test: participants tap on target responding to a visual queue (color shift). **(D)** A sample report summarizing test results.

Following minimal training by the site personnel, iMotor screens (Figure 1) guided participants to perform the following neurological tests:

(1) *Two-target finger tapping test*: participants were prompted to alternatingly tap with the index finger, as fast and as accurate as possible, the center of two concentric circles on the tablet screen.
(2) *Pronation–supination test*: participants were prompted to alternatingly tap their palmar and dorsal surface of their hand as fast as possible on the tablet screen.
(3) *Reaction time test*: participants were asked to tap a target as fast and as accurate as possible immediately after a visual queue (color shift) on the tablet screen.

The duration of each test was set for 30 s. The total duration of patient interface with the application was on average 5 min. Patients performed tests with their more affected upper limb (as assessed by investigator). HCs performed the tests with their dominant hand. Following completion of tasks, subjects were asked to complete a satisfaction survey on the user interface experience with iMotor. The satisfaction survey was adapted from the Feeling of Satisfaction with Inhaler survey (FSI-10). The FSI-10 is a validated, self-report instrument containing 10 questions, each requiring a response on a 5-point Likert scale (very, fairly, somewhat, not very, and hardly at all) scored from 5 to 1, respectively. The original instrument, designed for an inhaler, assesses the level of satisfaction with a device and includes items on ease or difficulty of use, portability, and usability (11). The adapted instrument is included as Supplementary Material.

Raw data were wirelessly transmitted real-time for algorithmic processing and storage to a HIPPA-compliant database. Hardware usability, software performance, and communication protocols were assessed.

iMotor recorded the screen pixel position (*x, y* coordinates) during finger-screen interface during the three-motor function tests (two-target finger tapping, pronation–supination, and reaction time) and algorithmically derived the following variables:

*Total number of taps*: number of finger/hand taps on the screen within the predetermined period of time (30 s).
*Tap accuracy (pixels)*: average number of pixels away from the target center and the SD of the average.
*Tap velocity (cm/s)*: the average distance the index finger “travels” between target A and B within a second [*v* = (*P*_B_ − *P*_A_)/*t*] and the SD of the average.
*Tap interval (ms)*: the average time between two consecutive finger (or hand) screen taps and the SD of the average.
*Tap duration (ms)*: the average time the index finger (or hand) touches the screen per tap during 30 s and the SD of the average. Calculated as the time difference between the hand touching and the hand leaving the screen per tap.
*Reaction time (ms)*: the average time the index finger needs to touch the screen following a visual stimulus generated by the application during 30 s and the SD of the average. A summary of variables (by test) is presented in Table S1 in the Supplementary Material.

Data were described using mean and SD by groups (HC and PD). Individual performance of tapping variables in differentiating groups was obtained using logistic regression analysis, and predictive performance was evaluated using the area under curve (AUC). Furthermore, the combined performance of tapping data was examined using a logistic regression model including various combinations of tapping variables. The model that provided maximum AUC and correct classification accuracy was selected as the best predictive model for PD compared to HV. Results of logistic regression analysis were summarized using odds ratio along with 95% confidence interval (CI) and *p*-value. Appropriate cutoff was determined using receiver operating characteristics curve analysis. The cutoff was selected where the model yielded maximum sensitivity (Se) and specificity (Sp). Pearson’s correlation coefficients (*r*) between MDS-UPDRS-III and tapping variables were also computed. All the analyses were carried out using STATA 12.1. All analyses are considered exploratory, and no corrections for multiple comparisons were applied. *p*-Values should be considered nominal. No formal sample size estimation was used. The final sample size was determined by our ability to accurately detect differences between disease and healthy states based on the performance characteristics of iMotor.

## Results

Thirty-eight participants (19 with PD and 17 HCs) were recruited in the study. This sample size was considered adequate for feasibility and detection of performance characteristics of iMotor and analytics efforts. PD subjects were 53% male, with a mean age of 67.8 years (±8.8), mean disease duration of 6.5 years (±4.6), MDS-UPDRS-III score 26.3 (±6.7), and H&Y stage 2. HCs were 47% male and had an age of 53.0 (±17.3) years. All subjects (HC and PD) were right handed and had at least a high school diploma. Application data were successfully recorded and transmitted to the study database. There was one tablet-related incident of hardware malfunction (failure to charge) that occurred while the tablet was idle.

### Univariate Analysis

In univariate analyses, the following tapping variables were significantly different in PD compared to HC: (A) two-target test: (1) total taps, (2) velocity, and (3) average interval; (B) pronation–supination test: (1) total taps; and (C) reaction time test: (1) reaction time. Conversely, the following tapping variables were not significantly different between PD and HC: (A) two-target test: (1) tap accuracy, (B) pronation–supination test: (1) tap duration, and (C) reaction time test: (1) accuracy. Reaction time variable provided the highest predictive ability for PD (90%) (Table 1). As expected, PD subjects recorded a lower number of finger and pronation–supination taps with higher intertap interval and slower tap velocity and reaction times.

**Table 1:** Summary of significant results (univariate analysis).

Variable measured	PD, mean(SD)	HC, mean(SD)	*p*-Value
Two-target total taps	100.6 (21)	131.4 (18.3)	0.003
Two-target tapping velocity (cm/s)	15 (3.2)	19.3 (2.9)	0.004
Two-target average interval (ms)	313 (74.7)	238.2 (35.9)	0.006
P/S total taps	75.7 (12.2)	95.2 (17.5)	0.005
P/S tapping average interval (ms)	406.4 (73.6)	328.7 (68.8)	0.01
Reaction time (ms)	89.4 (23.1)	54.5 (18.3)	0.004
Reaction accuracy	28.5(9.5)	24.7(5.2)	0.167

*SD, standard deviation*.

### Multivariable Analysis

In the multivariable logistic regression model, reaction time (reaction time test) (*p* = 0.021) and total taps (two-target test) (*p* = 0.026) were associated with PD. In addition, a borderline association between two-target accuracy and PD was observed (*p* = 0.056). MDS-UPDRS-III was negatively correlated with two-target tap accuracy (*r* = −0.35) and pronation–supination tap interval (*r* = −0.45) and positively correlated with pronation–supination total taps (*r* = 0.45). The combined performance of tapping variables in PD versus HCs is presented in Table S2 in the Supplementary Material.

### AUC Analysis

The model with reaction time provided highest predictive ability (AUC = 90%) followed by the model with two-target total taps (AUC = 86%), two-target interval (AUC = 83%), and two-target tap velocity (AUC = 83%) The combined model with two-target total taps, reaction time, and two-target average accuracy produced maximum discriminatory performance between HC and PD. The overall accuracy (AUC) of the combined model was obtained as 0.98 (95% CI: 0.93–1). The cutoff for the developed predictive model was determined as ≥0.78 with sensitivity (94%), specificity (93%), overall correct classification (94%), likelihood ratio positive (12), and likelihood ratio negative (0.06).

### Patient Satisfaction Survey

Most patients considered the iMotor tests simple and easy (79% very easy, 16% fairly easy, and 5% somewhat easy) to perform. PD subjects were willing to continue using iMotor at home to monitor their disease (63% very willing and 21% fairly willing). All subjects agreed that they would repeatedly use iMotor to improve motor performance if their doctor recommended it. Many (79%) subjects viewed iMotor as a fun passing activity (gaming activity). Ninety-five percent of study participants were very interested in comparing their results with those of other subjects with PD. Full results of the survey are available in the Supplementary Material.

## Discussion

In this feasibility study, we demonstrated that iMotor could be deployed in a clinical setting and could be used by site personnel and PD patients without technical difficulties. While further studies are required to validate our findings, results from this cross-sectional, exploratory study suggest that iMotor objectively differentiates individuals with PD from HCs using data captured through smart device sensing technology while users perform tests routinely used in clinical practice. iMotor’s technical performance feasibility testing, including hardware positioning, usability, software performance, and communication protocols, revealed no issues suggesting capabilities for future scale-up and following further validation, longitudinal deployment as a marker of motor function.

Progressive reduction in finger tapping amplitude, speed or their combination, critical to PD, may not be adequately assessed by clinical scales (13). However, clinicians may not detect subtle and mixed changes in amplitude, accuracy, velocity, and rhythm of rapid finger movement (14). Using sensor TOMs (accelerometry, gyrometry, and touch screens) to quantify clinical observations, such as slowness in repetitive hand movements and reaction time, could have a profound effect in research and patient care. Quantitative portable measurements are easier to administer and may reduce the need for in-clinic visits. Unlike clinical rating scales that utilize categorical ratings, objective symptom monitoring can quantify motor scores on a continuum, allowing for greater precision in recording subtle changes in PD motor symptomatology (5).

Several studies have demonstrated that remote monitoring systems and virtual visits improve the quality of care while minimizing direct and indirect health-care costs (12). Introduction of simple, reliable, and sensitive objective measures, particularly applicable in home environments, to supplement the in-office clinical evaluation has the potential to enhance the management of PD (1).

Patient satisfaction is directly linked to patient engagement and improved health outcomes (15). To date, the main focus of new technology’s validation process is limited to collection of big data for analytic purposes often neglecting to capture patient perspectives. The applicability of technology-based objective measures in both clinical research and everyday care is highly dependent on long-term patient engagement and satisfaction. Therefore, the high rates of patient satisfaction observed combined with the potential for sensitive monitoring of motor function, favorably position iMotor as a TOM for PD in a patient-centric, value-driven health-care environment. The high satisfaction rate values confirm that a smart tablet-based application could be used at home and could reduce the in-clinic visit need.

Limitations of this feasibility study include its exploratory, uncontrolled nature, the small sample size, the cross-sectional design, and the younger age of the HC cohort. Our study only focused on hand movements and reaction time. Additional objective measures, such as gait and voice processing, may more comprehensively reflect overall functional state. For comprehensive evaluation and management of PD, in addition to motor symptoms, non-motor symptoms should be monitored using similar technologies through smart tablet applications.

Given the promising feasibility results, iMotor will require further validation for remote PD monitoring in longitudinal, controlled clinical trials. In addition, evaluation of iMotor for preclinical monitoring, early diagnosis, and optimization of therapies is being planned. The combination of remote monitoring and report integration may lead to improved PD patient outcomes by enhancing the decision-making process and optimizing management plans with on-time, personalized drug dosage modifications.

## Ethics Statement

Participants gave informed, written consent for this study. All procedures were approved by the University of Cincinnati Institutional Review Board.

## Author Contributions

GM: research project: conception and organization; statistical analysis: design, execution, and review and critique; and manuscript: writing of the first draft and review and critique. EM, IT, and AS: research project: organization and execution; and manuscript: review and critique. BDW: research project: conception; statistical analysis: design; and manuscript: review and critique. EB: statistical analysis: design and execution; and manuscript: review and critique. AD: statistical analysis: execution; and manuscript: review and critique. AE: research project: conception; and manuscript: review and critique. SP: research project: conception; and statistical design: review and critique; and manuscript: review and critique.

## Conflict of Interest Statement

With the exception of GM and SP, none of the investigators have a financial interest in iMotor or Apptomics Inc.
