# Supplementary material for: Biometric Digital Health Technology for Measuring Motor Function in Parkinson’s Disease: Results from a Feasibility and Patient Satisfaction Study
Source: Front Neurol. 2017 Jun 13;8:273. doi: 10.3389/fneur.2017.00273 (PMC5468407; doi:10.3389/fneur.2017.00273)
Supplement: Supplementary file 1 [file table_1.docx]

**STable 1.** Summary of iMotor variables (by test)

| **Variable measured** | **Two-target test** | **Pronation-Supination test (P/S)** | **Reaction time test** |
| --- | --- | --- | --- |
| Total Taps | ✓ | ✓ |  |
| Tap velocity | ✓ |  |  |
| Tap interval | ✓ | ✓ |  |
| Tap duration | ✓ | ✓ |  |
| Tap accuracy | ✓ |  |  |
| Reaction time |  |  | ✓ |
