# Supplementary material for: Biometric Digital Health Technology for Measuring Motor Function in Parkinson’s Disease: Results from a Feasibility and Patient Satisfaction Study
Source: Front Neurol. 2017 Jun 13;8:273. doi: 10.3389/fneur.2017.00273 (PMC5468407; doi:10.3389/fneur.2017.00273)
Supplement: Supplementary file 2 [file table_2.docx]

STable 2. Combined performance of tapping variables in PD versus HCs

| **Variable**  **Measured** | **OR** | **95%CI** | | **p-value** |
| --- | --- | --- | --- | --- |
| Two-target  total taps | 0.83 | 0.70 | 0.98 | 0.026 |
| Two-target Accuracy | 1.25 | 1.00 | 1.56 | 0.056 |
| Reaction time | 1.21 | 1.03 | 1.43 | 0.021 |
| OR: odds ratio; CI: confidence interval; Probability of PD= exp (0.30 -0.19* 2-Target -Total Taps  +0.19*Reaction time- +0.22* 2-Target - Average Accuracy)/(1+ exp(0.30  -0.19* 2-Target -Total Taps  +0.19*Reaction time +0.22* 2-Target - Average Accuracy) | | | | |
