# Supplementary figures and images for: Biometric Digital Health Technology for Measuring Motor Function in Parkinson’s Disease: Results from a Feasibility and Patient Satisfaction Study
Source: Front Neurol. 2017 Jun 13;8:273. doi: 10.3389/fneur.2017.00273 (PMC5468407; doi:10.3389/fneur.2017.00273)

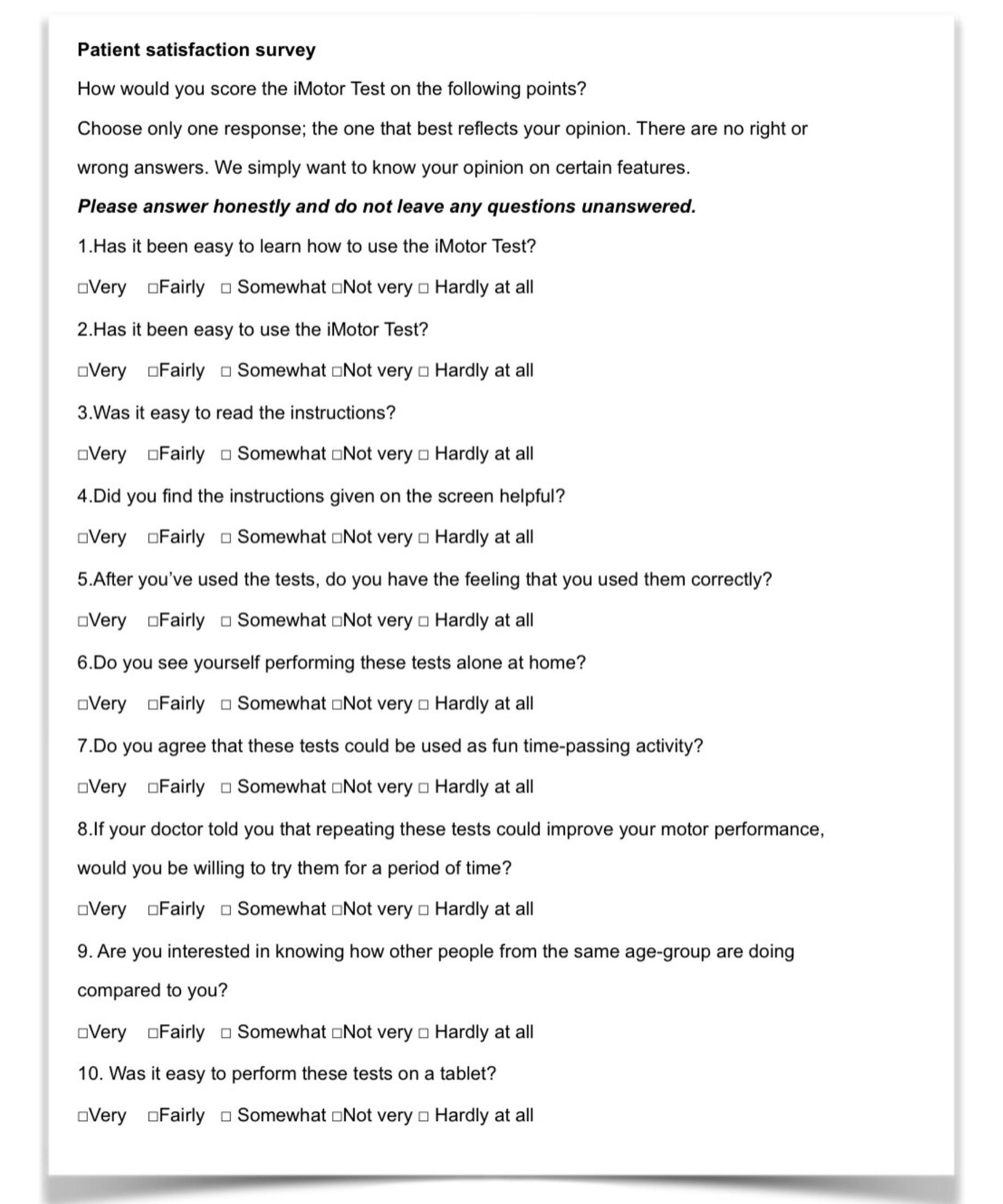

Supplement: Supplementary file 3 [file image_1.tif]

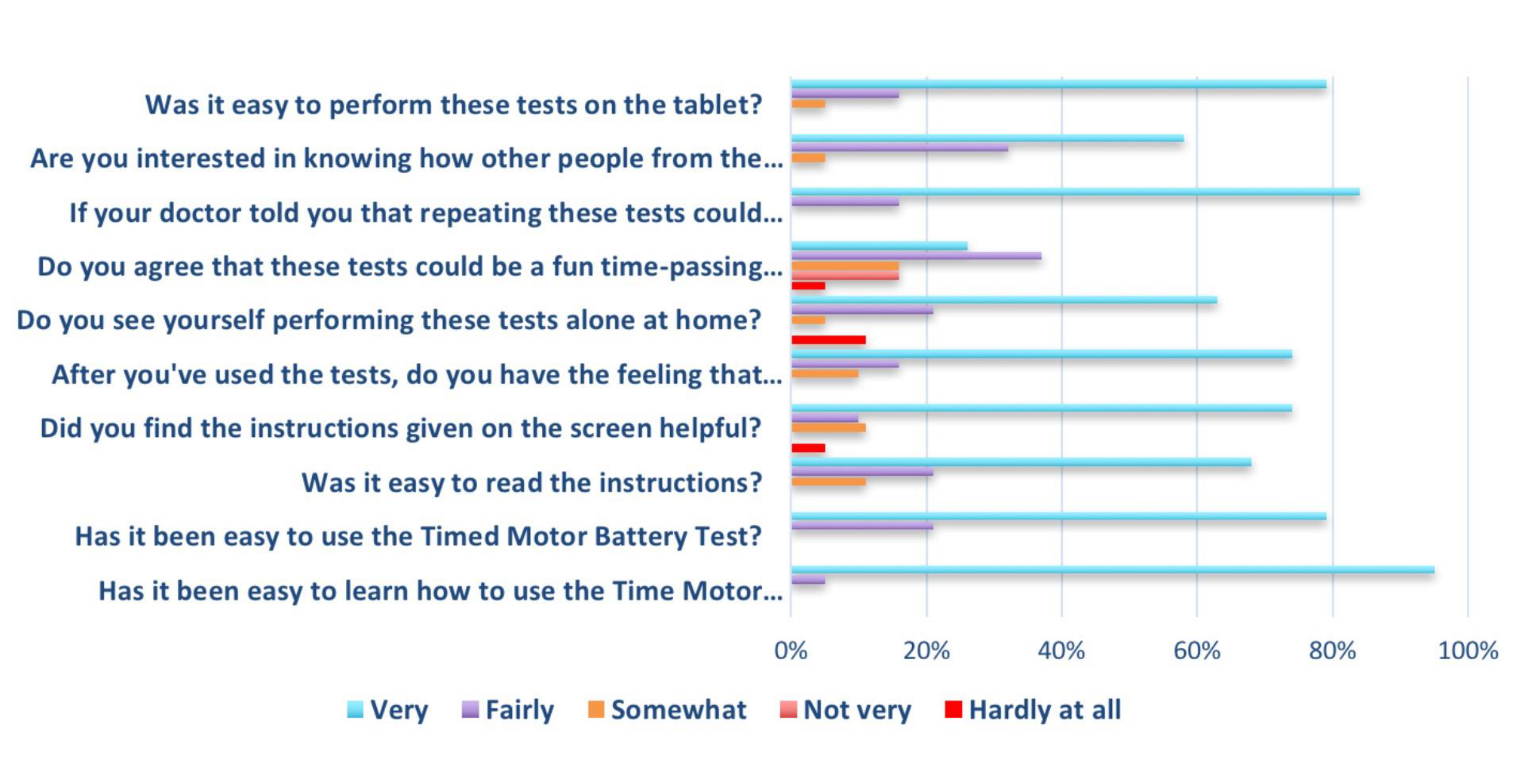

Supplement: Supplementary file 4 [file image_2.tif]
